# Impaired grouping of ambient facial images in autism

**DOI:** 10.1038/s41598-022-10630-0

**Published:** 2022-04-23

**Authors:** Bayparvah Kaur Gehdu, Katie L. H. Gray, Richard Cook

**Affiliations:** 1 Department of Psychological Sciences, Birkbeck, University of London, Malet Street, London, WC1E 7HX UK; 2 School of Psychology and Clinical Language Sciences, University of Reading, Reading, UK; 3 Department of Psychology, University of York, York, UK

**Keywords:** Psychology, Human behaviour

## Abstract

Ambient facial images depict individuals from a variety of viewing angles, with a range of poses and expressions, under different lighting conditions. Exposure to ambient images is thought to help observers form robust representations of the individuals depicted. Previous results suggest that autistic people may derive less benefit from exposure to this exemplar variation than non-autistic people. To date, however, it remains unclear *why*. One possibility is that autistic individuals possess atypical perceptual learning mechanisms. Alternatively, however, the learning mechanisms may be intact, but receive low-quality perceptual input from face encoding processes. To examine this second possibility, we investigated whether autistic people are less able to group ambient images of unfamiliar individuals based on their identity. Participants were asked to identify which of four ambient images depicted an oddball identity. Each trial assessed the grouping of different facial identities, thereby preventing face learning across trials. As such, the task assessed participants’ ability to group ambient images of unfamiliar people. In two experiments we found that matched non-autistic controls correctly identified the oddball identities more often than our autistic participants. These results imply that poor face learning from variation by autistic individuals may well be attributable to low-quality perceptual input, not aberrant learning mechanisms.

## Introduction

Autism spectrum disorder (hereafter autism) is a neurodevelopmental condition associated with social communication difficulties, together with restricted and repetitive patterns of behaviours and intensive interests^1^. The presence of face recognition problems does not form part of the diagnostic criteria for autism. Nevertheless, it is well-established that many people with autism find face identification challenging^2,3^. In order to fully understand the impact of these problems on autistic individuals, it is important to establish the nature and extent of deficits, where observed. To date, however, the face processing difficulties seen in autism remain poorly understood.

According to one influential review, the structural encoding of faces is intact in autism^2^. Rather, autistic individuals are thought to have a selective face memory deficit that hinders their ability to retain facial percepts in visual memory. Contrary to this view, however, recent findings suggest that early structural encoding of faces is impaired in autism^4,5^. For example, autistic participants show deficits on standardised tests of face perception that have minimal memory demands^4^ and exhibit reduced sensitivity to (some) facial illusions^5^.

Many other questions also remain unresolved. For example, there is considerable disagreement within the literature surrounding the extent to which autistic individuals process faces holistically^5–7^ and whether individuals exhibit aberrant facial adaptation^8,9^. The propensity of autistic individuals to orient their attention to faces is also debated^10,11^. Similarly, studies of expression recognition in autism have yielded inconsistent findings^12,13^.

Neuroimaging has revealed several regions that appear to contribute to the visual analysis of faces, including the occipital^14^ and fusiform face areas^15^. A considerable body of evidence suggests that these areas exhibit weaker responses when autistic individuals view faces, relative to the activations seen in non-autistic participants^16–20^. There is also some suggestion that the N170—an event-related potential measured using electroencephalography (EEG) that appears ~ 170 ms after the onset of a face stimulus^21,22^—is delayed in many autistic participants, suggestive of less efficient face processing^23^.

The present study sought to establish whether autistic people are less able to group ambient facial images by the identity of those depicted. Ambient images are naturalistic images of faces that depict individuals from a variety of viewing angles, with a range of expressions, under different lighting conditions. There has been a great deal of interest in the visual processing of ambient images. Crucially, the exemplar variation present within ambient images is thought to help individuals form “robust” representations of the individuals depicted^24,25^. Once acquired, robust representations support the seemingly effortless recognition of familiar faces. Until a robust representation has been acquired, the identification and matching of unfamiliar faces remains effortful and inaccurate^26^. Representations are said to be ‘robust’ insofar as they are relatively insensitive to image specific variation; for example, robust representations support identification, despite salient changes in lighting and pose.

The nature of the resulting robust representations remains unclear. According to one perspective, the visual system derives a perceptual average from different exemplars of the same face, that is easy to match to new instances encountered subsequently^27^. Alternatively, each separate encounter with a face may be stored, and familiar faces are recognised through comparison with previously stored instances^28^. Having encountered a given face on many occasions, in different poses, lighting and viewing conditions, observers are able to densely sample the potential instance space. Thereafter, the likelihood of a close match between a novel encounter and a previously stored instance is high, yielding superior recognition performance. According to this view, robust representations can be thought of as a comprehensive “instance database”.

Ipser and colleagues^29^ previously reported that, relative to non-autistic controls (*N* = 20), autistic participants (*N* = 20) were less able to form robust representations of particular facial identities from ambient images. During a training procedure, participants learned eight facial identities by viewing ambient images (96 of each to-be-learned identity). At test, participants were shown a set of new exemplars, half of which depicted the learned facial identities and half of which depicted novel facial identities. Compared with non-autistic controls (74.7% correct), the autistic participants (62.2% correct) were less able to identify the individuals encountered during the study phase^29^.

To date, however, it remains unclear *why* autistic individuals are less able to form robust representations from ambient images. Ipser and colleagues^29^ speculated that autistic people may possess atypical perceptual learning (or “exemplar pooling”) mechanisms. For example, they may be less able to derive a perceptual average from multiple exemplars than non-autistic participants. However, a second possibility is that autistic individuals have a more fundamental problem that affects the perceptual encoding of the people depicted in ambient images; i.e., the learning mechanism may be intact, but receive low-quality input from face encoding processes. This possibility has recently gained support from reports that autistic individuals struggle on face matching tasks with low memory demands^4^.

To test this hypothesis, we examined whether autistic people are less able to group ambient images of unfamiliar individuals based on their identity. Every trial depicted a novel combination of individuals, thereby ensuring that participants had little or no opportunity for perceptual learning across trials. As such, the task was intended to be a pure measure of participants’ ability to group ambient images of unfamiliar people in the absence of any face learning and robust representation. Crucially, the variation present within the ambient images was very similar to that present in the images employed by Ipser and colleagues^29^. Should autistic participants be impaired on this task, it would suggest that previous evidence of poor face learning from variation^29^, may be attributable to low-quality perceptual input, not aberrant learning mechanisms.

## Experiment 1

### Participants

Sixty participants with a clinical diagnosis of autism (*M*_age_ = 32.75 years; *SD*_age_ = 11.25 years) were recruited via www.ukautismresearch.org. All of the autistic participants exhibited typical levels of intelligence and verbal ability. Of the 22 individuals who described their sex as male, 16 described their gender identity as male, 4 identified as non-binary, 1 identified as female, and 1 preferred not to state their gender identity. Of the 38 individuals who described their sex as female, 28 described their gender identity as female, 7 identified as non-binary, and 3 identified as male. All autistic participants had received an autism diagnosis (e.g., Autism Spectrum Disorder, Asperger’s Syndrome) from a clinical professional (General practitioner, Neurologist, Psychiatrist, or Clinical Psychologist) based in the UK. All participants in the autistic group also reached cut-off (a score of 32) on the Autism Spectrum Quotient (AQ; Baron-Cohen et al., 2001). The mean AQ score of the autistic group was 41.27 (*SD* = 4.26).

Sixty non-autistic individuals (*M*_age_ = 33.63 years; *SD*_age_ = 7.99 years) were recruited through www.prolific.co to serve as controls. Of the 60 participants in the non-autistic group, 25 described their sex and gender identity as male and 35 described their sex and gender identity as female. All non-autistic participants scored below cut-off (a score of 31 or less) on the AQ. The mean AQ score of the non-autistic group was 18.18 (*SD* = 6.77).

To be eligible, all participants (autistic and non-autistic) had to be aged between 18 and 60, had to speak English as a first language, and had to be a current UK resident. The autistic and non-autistic participants did not differ significantly in terms of participants’ age [*t*(118) = 0.496, *p* = 0.621] or sex [*X*^*2*^_(1)_ = 0.315, *p* = 0.575]. However, the groups did differ in terms of participants’ gender identity [*X*^*2*^_(2)_ = 13.381 *p* = 0.004].

In addition to the AQ, all participants completed the 20-Item Prosopagnosia Index (PI20)^30–32^; a self-report measure of the traits associated with developmental prosopagnosia (DP). DP is a neurodevelopmental condition characterised by severe face recognition impairments that is thought to co-occur with autism^33,34^. Consistent with previous reports^4,35^, the PI20 scores of the autistic participants (*M* = 65.20, *SD* = 15.28, range 30–93) were significantly higher than those of the non-autistic controls (*M* = 46.83, *SD* = 12.28, range 26–75) [*t*(118) = 7.258, *p* < 0.001].

All participants also completed a measure of abstract visuospatial reasoning. Forty items were selected from The Matrix Reasoning Item Bank (MaRs-IB)^36^. Participants were given 30 s to complete each puzzle by selecting the correct answer from 4 options. Participants responded using keyboard number keys (1–4). Participants were given a 5 s warning before the end of each trial. No feedback was given during the test. All participants attempted all forty items. Participants had to complete 3 practice trials correctly before beginning the test. The scores of the autistic participants (*M* = 25.62, *SD* = 5.72, range 14–37) and the non-autistic controls (*M* = 26.48, *SD* = 5.42, range 13–37) did not differ significantly [*t*(118) = 0.852, *p* = 0.396].

Autistic participants also completed the Interpersonal Reactivity Index (IRI)^37^, and the Toronto Alexithymia Scale (TAS)^38^. Full details of the autistic sample are provided as Supplementary Material. Ethical clearance was granted by the local ethics committee (the Departmental Ethics Committee for Psychological Sciences, Birkbeck, University of London) and the experiment was conducted in line with the ethical guidelines laid down in the 6th (2008) Declaration of Helsinki. All participants gave informed consent before taking part.

### Experimental task

**Figure 1:**
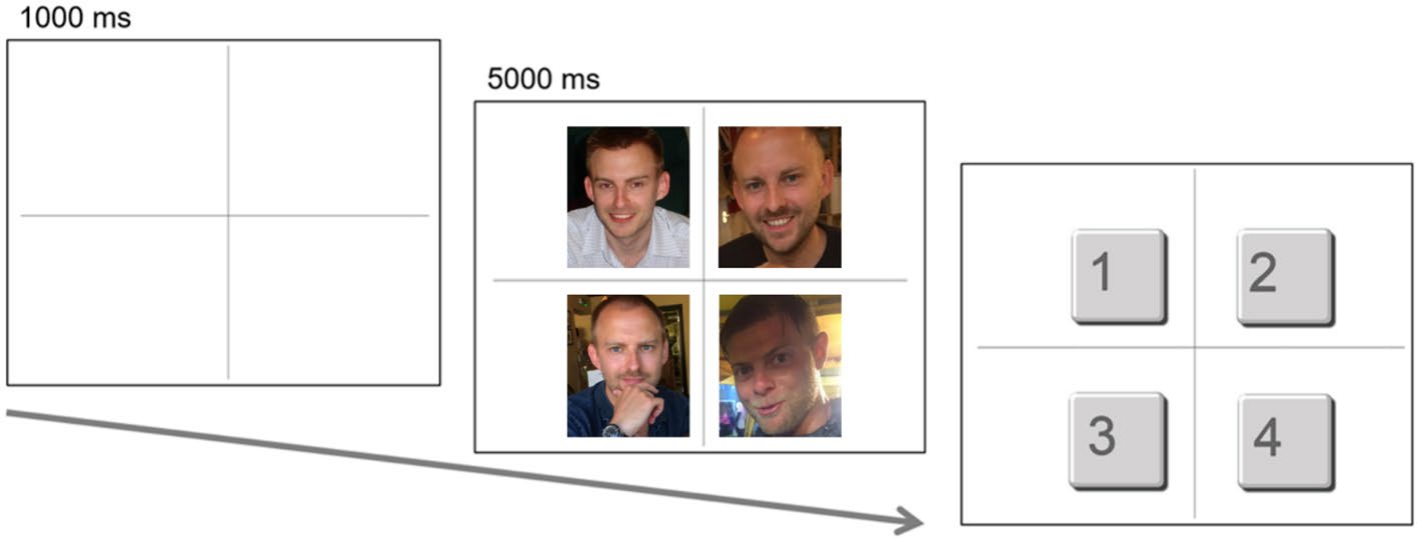
Schematic illustration of an experimental trial. The facial images shown in the figure were not used in the study, but are representative of the ambient images employed. The images shown are owned by the authors. The individuals depicted have given informed consent for the open-access publication of the images.

Trials began when a large cross appeared, dividing the participants’ display into four quadrants. After 1000 ms, four ambient images appeared on the display, one in each quadrant. On each trial, three of the ambient images depicted the same person. The fourth ‘oddball’ image depicted a different person of broadly similar appearance. The four ambient images were presented for 5000 ms. During this time, participants were asked to identify which of the four images was the oddball. After 5000 ms, the ambient images disappeared and were replaced with the response screen. Participants indicated which of the four images was the oddball by pressing the corresponding number key (Fig. 1). This approach ensured that all participants inspected the to-be-judged images for the same length of time, thereby mitigating any speed-accuracy trade-off.

Participants completed 40 experimental trials in a randomised order. Twenty trials depicted White females; 20 trials depicted White males). Participants were invited to take a short break after 10, 20, and 30 trials. At the end of the procedure, participants were asked if they recognised any of the people depicted. None of the faces were recognised. In total, the experiment required 160 ambient images (80 male, 80 female) that were sourced online from various websites.

Before the task started, participants completed 3 practice trials to ensure they understood what the task required. Practice trials had the same format as the experimental trials; however, images of cartoon characters were used instead of photographic ambient images. Participants had to get all practice trials correct before progressing to the experimental trials. Four catch trials were interspersed within the experimental trials. The catch trials had an identical format to the practice trials.

The experiment was conducted online using Gorilla Experiment Builder^39^. A calibration procedure at the start of the experiment ensured that each ambient image appeared 5 cm high, positioned centrally within each quadrant, irrespective of the particular dimensions of each participant’s monitor. The experiment had to be completed on a desktop or laptop computer; it would not run on a mobile device or tablet. The experimental tasks are available as Open Materials at gorilla.sc (https://app.gorilla.sc/openmaterials/332894).

### Results

The ability of the two groups to identify the oddball images was assessed through an independent-samples *t*-test (α = 0.05, two-tailed). Correlations were assessed by computing Pearson correlation coefficients (α = 0.05, two-tailed). The data supporting all of the analyses described are available via the Open Science Framework (https://osf.io/fj7de/).

**Figure 2:**
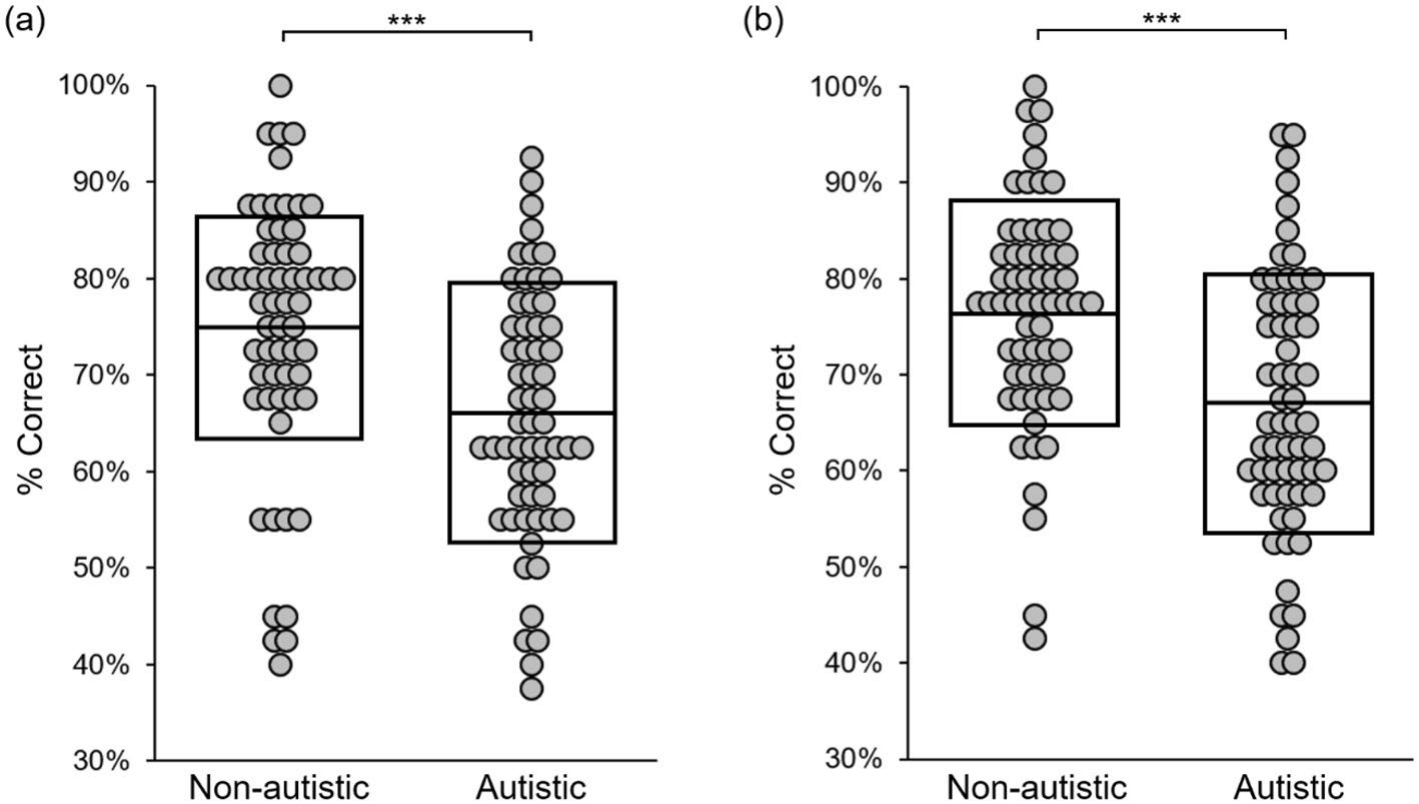
Results from Experiment 1 (**a**) and Experiment 2 (**b**). Boxes indicate group mean ± SD. ***denotes significance at *p* < .001.

Autistic participants (*M* = 65.96% *SD* = 12.72%) correctly identified fewer oddball images than the non-autistic controls (*M* = 74.71% *SD* = 13.74%) [*t*(118) = 3.62, *p* < 0.001, *d* = 0.661] (Fig. 2a). All participants responded correctly on at least 3 of the 4 catch trials.

In the combined sample (*N* = 120), significant correlations were seen between participants’ AQ scores (*r* = −0.276, *p* = 0.002) and PI20 scores (*r* = −0.374, *p* < 0.001) and their % correct achieved on the experimental task. Separate hierarchical regression analyses revealed that PI20 scores [*β* = −0.287, *t* = 2.809, *p* = 0.006] but not AQ scores [*β* = 0.044, *t* = 0.221, *p* = 0.826] were predictive of task performance once the effect of Group (autistic, non-autistic) [*β* = −0.316, *t* = 3.62, *p* < 0.001] was removed. No correlation was seen between task performance (% correct) and participants’ scores on the abstract reasoning task (*r* = 0.170, *p* = 0.064).

In the autistic sample (*N* = 60), a significant correlation was seen between task performance (% correct) and participants’ PI20 scores (*r* = −0.279, *p* = 0.031). The correlations between % correct and AQ scores (*r* = −0.157, *p* = 0.232), TAS-20 scores (*r* = −0.188, *p* = 0.151), and IRI scores (*r* = 0.243, *p* = 0.062) were all non-significant.

## Experiment 2

The results from our first experiment suggest that autistic people are less able to group ambient images according to the identity of the people depicted. Our second experiment was a replication of the first. Importantly, however, we used an entirely different set of ambient images in order to confirm that the group difference seen in Experiment 1 was not attributable to the particular images used. By definition, ambient images are uncontrolled. For example, faces are shown at different scales, with different poses and expressions. Similarly, the images are captured using different cameras, at different distances, under different lighting conditions. Because of the random variation present within sets of ambient images, the danger that effects are due to idiosyncratic stimulus features, is greater than when using facial images drawn from standardised databases^40–42^. It is therefore important to confirm that effects generalise to different image sets.

The new set of 160 images (80 male, 80 female) were sourced in the same manner as the first set. Once again, the variation present within the ambient images was similar to that present in the set employed by Ipser and colleagues^29^. With the exception of the images used, the methods of Experiment 1 and 2 were identical.

### Participants

The same sixty autistic participants completed the second experiment. The autistic participants completed the first and second experiments during separate testing sessions. We were unable to contact and recruit the same group of non-autistic controls that completed Experiment 1. We therefore recruited a new group of 60 non-autistic controls (*M*_age_ = 30.75 years; *SD*_age_ = 7.5 years) through www.prolific.co.

Of the 60 participants in the non-autistic group, 30 described their sex and gender identity as male and 28 described their sex and gender identity as female. Two participants preferred not to reveal their sex and gender identity. All non-autistic participants scored below cut-off (a score of 31 or less) on the AQ. The mean AQ score of the non-autistic group was 17.65 (*SD* = 7.3). Once again, all participants had to be aged between 18 and 60, had to speak English as a first language, and had to be a current UK resident.

The autistic and non-autistic participants did not differ significantly in terms of age [*t*(118) = 1.146, *p* = 0.254 0.455] or sex [*X*^*2*^_(1)_ = 4.746, *p* = 0.093]. However, the group did differ in terms of gender identity [*X*^*2*^_(2)_ = 13.417, *p* = 0.004]. The PI20 scores of the autistic participants (*M* = 65.20, *SD* = 15.28, range 30–93) were significantly higher than those of the non-autistic controls (*M* = 42.55, *SD* = 11.96, range 24–72) [*t*(118) = 9.041, *p* < 0.001]. The scores of the autistic (*M* = 25.62, *SD* = 5.72, range 14–37) and non-autistic participants (*M* = 24.93, *SD* = 6.51, range 13–36) on the abstract reasoning task did not differ significantly [*t*(118) = 0.611, *p* = 0.543].

### Results

As in the first experiment, the autistic participants (*M* = 66.92% *SD* = 13.54%) correctly identified fewer oddball images than the non-autistic controls (*M* = 76.67% *SD* = 11.48%) [*t*(118) = 4.255, *p* < 0.001, d = 0.777] (Fig. 2b). All participants responded correctly on at least 3 of the 4 catch trials.

In the combined sample (*N* = 120), significant correlations were seen between participants’ AQ scores (*r* = −0.405, *p* < 0.001) and their PI20 scores (*r* = −0.287, *p* = 0.001) and their % correct achieved on the experimental task. Separate hierarchical regression analyses revealed that AQ scores [*β* = −0.392, *t* = 2.079, *p* = 0.040], but not PI20 scores [*β* = −0.085, *t* = 0.761, *p* = 0.448] were predictive once the effects of Group (autistic, non-autistic) [*β* = −0.365, *t* = 4.255, *p* < 0.001] were removed. No correlation was seen between task performance (% correct) and participants’ scores on the abstract reasoning task (*r* = −0.025, *p* = 0.789).

In the autistic sample (*N* = 60), the correlations between % correct and participants’ AQ scores (*r* = −0.116, *p* = 0.379), PI20 scores (*r* = 0.066, *p* = 0.617), TAS-20 scores (*r* = −0.181, *p* = 0.165), and IRI scores (*r* = 0.144, *p* = 0.273) were all non-significant.

## General discussion

In the present study, we conducted two experiments to assess the ability of autistic participants to group so-called ambient images by the identity of the people depicted. In order to correctly group individuals depicted in ambient images, participants must identify commonalities in facial structure across instances and disregard image-specific variation (e.g., differences in pose and lighting). In both experiments, participants were shown arrays of four ambient images for 5000 ms. Each array contained 3 images of one person and a single image of a different person. Participants were tasked with finding the oddball facial image within each array. In both experiments, autistic participants (*N* = 60) found this task more challenging—they were less accurate—than matched non-autistic controls (*N* = 60).

### Implications for face learning in autism

Exposure to the facial variation present in ambient images is thought to facilitate face learning^24,25,27,43^. Seeing to-be-learned individuals in a variety of poses appears to help observers form an accurate representation of their facial appearance that helps them identify that person in subsequently encountered instances. Robust representations may take the form of an average^27^ or a comprehensive database of previously encountered instances^28^.

Previous research has shown that autistic individuals derive less benefit from facial variation than non-autistic controls when learning faces^29^. However, in light of the present findings, we suggest that what may superficially appear to be evidence of aberrant face learning, in fact reflects poor quality input into perceptual learning mechanisms. For example, if the perceptual description of individual exemplars is noisy and imprecise, this will make it harder for the visual system to derive a high-quality average of the instances encountered^27^. The resulting person-specific averages may be less distinctive than the equivalent representations derived by neurotypical observers.

A further intriguing possibility is that poor face encoding may cause autistic participants to make “sorting errors”. In order to acquire robust representations of to-be-learned facial identities—say Matt Damon and Brad Pitt—the visual system must somehow group the instances of Matt Damon together, and the instances of Brad Pitt together. Having been sorted, instances can be combined to form an average representation^27^ or pooled in an instance database^28^. However, if instances of Matt Damon are erroneously tagged as Brad Pitt, the robust representation of Brad Pitt will be derived from a mix of instances depicting Brad Pitt and Matt Damon. Such a representation would likely hinder recognition of Brad Pitt in subsequently encountered instances.

Impaired perceptual encoding of faces provides a single parsimonious explanation of the present results and those described by Ipser and colleagues^29^; i.e., there is no need to hypothesise an additional deficit that affects face learning. In principle, however, autistic people may still exhibit a second deficit that affects face learning—our results do not rule this out. It may prove difficult to evidence convincingly an additional face learning deficit in autistic participants who show impaired perceptual encoding of novel unfamiliar faces. However, if putative deficits of face learning and perceptual encoding can occur independently, autistic individuals may exist who show typical face encoding, but aberrant face learning.

### The nature of face processing deficits in autism

In 2012, an influential systematic review concluded that autistic people form accurate perceptual descriptions of faces, but struggle to retain facial percepts in visual memory for more than a few seconds^2^. Thus, face identification difficulties, where observed might be a product of aberrant short-term face memory, not poor perceptual encoding. Together with recent results^4^, our findings challenge this conclusion. The task used in the present study had extremely low memory demands. Specifically, because all four faces were presented simultaneously, participants had little need to retain face percepts in visual memory. Nevertheless, autistic participants performed less accurately relative to non-autistic controls. In light of these findings, it seems increasingly likely that face encoding is impaired in this population.

It has been suggested previously that the face recognition problems seen in autism might be well-characterised as co-occurring DP—a different neurodevelopmental condition associated with severe lifelong face recognition problems^33,44^. Many neurodevelopmental conditions occur with a greater incidence in the autistic population than in the general population, including attention deficit and hyperactivity disorder^45^, developmental coordination disorder^46^, developmental alexithymia^47^, specific language impairment^48^, dyslexia^49^, and synaesthesia^50^. Given that the co-occurrence of neurodevelopmental disorders is ‘the norm’ rather than ‘the exception’ it would be surprising if there was not an elevated rate of DP in the autistic population.

It is clear that autism and DP are independent conditions. Many people present with DP without signs of autism^51^. Conversely, many autistic individuals also perform typically on face recognition tasks^4,52^. Nevertheless, many autistic individuals experience debilitating face recognition difficulties^53^. We saw evidence of this heterogeneity in our data—while some autistic participants struggled with the task, others achieved near-perfect levels of performance. This variability is precisely what one might expect if autism and DP were independent neurodevelopmental conditions, with a high level of co-occurrence^33,44^.

In this context, it is useful to ask whether the face identification problems seen in autism resemble those seen in DP. It is noteworthy that individuals with DP perform poorly on face identification tasks with minimal memory demands, suggestive of impaired face encoding. For example, DPs perform poorly on the Cambridge Face Perception Test, in which participants are asked to sort six simultaneously presented test faces, according to their resemblance to a target face^54,55^. Should the face recognition problems seen in autism reflect a problem with short-term face memory^2^, one could argue that different types of face processing deficit are seen in autism and DP. However, evidence of impaired face encoding observed here and elsewhere^4^ suggests that the face processing deficits in autism *do* resemble those seen in DP.

It is also of interest that many of our autistic participants (30 out of 60; 50%) scored above the cut-off for DP on the PI20. By comparison, far fewer non-autistic controls (12 out of 120; 10%) reached this cut-off. Similar findings have been reported elsewhere^4,35^. This questionnaire was designed to measure the traits associated with DP. The items were based on the qualitative experiences of individuals with this condition (e.g., When people change their hairstyle, or wear hats, I have problems recognizing them; I sometimes find movies hard to follow because of difficulties recognizing characters). The fact that many autistic individuals are scoring above cut-off suggests that they recognise the experiences described and are responding as though they have DP.

### Limitations

The present study was conducted online, an approach that is increasingly common. Carefully-designed online tests of cognitive and perceptual processing can yield high-quality data, indistinguishable from that collected in the lab^56–58^. To give recent examples from our own research, we have found that online testing has produced clear, replicable results in visual search and attention cueing experiments^59–62^, and studies of visual illusions^63,64^. However, this approach also has some well-known limitations. For example, it is not easy to control the testing environment, participants’ viewing distance, or their monitor settings.

A further limitation of the present work is the lack of diversity within our autistic sample and our face stimuli. The overwhelming majority (56 out of 60) of our autistic participants identified as White (typically White-British). For this reason, we opted to use facial stimuli that also depicted White individuals. This choice ensured that face processing impairments, where observed, could not be attributed to so-called ‘cross-race’ effects, whereby participants sometimes experience perceptual difficulties when viewing types of faces with which they are less familiar^65–67^. As such, however, it remains unclear how well our findings generalise to faces of other ethnicities and more diverse autistic populations.

Finally, while the same group of autistic participants completed both experiments, different non-autistic controls completed Experiments 1 and 2. This was necessary because we were unable to recruit the same 60 controls for Experiment 2. This feature raises the possibility that the autistic participants benefited from practice effects in Experiment 2, while the non-autistic controls did not. We note, however, that the pattern of results seen in the second experiment was very similar to that seen in the first; indeed, the effect size seen in Experiment 2 (*d* = 0.777) was numerically larger than that seen in Experiment 1 (*d* = 0.661). Given that the non-autistic participants achieved higher levels of accuracy than the autistic participants, it seems unlikely that differential practice effects can account for the group difference observed.

### Conclusion

The results of our two experiments suggest that autistic people are less able to group ambient facial images according to the identities of those depicted. Consistent with recent findings^4^, these results indicate that autistic individuals perform poorly on face identification tasks with minimal memory demands, suggestive of impaired face encoding. It has previously been shown that autistic people derive less benefit from facial variability when learning new facial identities^29^. The present findings suggest that this may well reflect poor perceptual input into learning mechanisms, not aberrant perceptual learning per se.

## Supplementary Information

Supplementary Information.

## Data Availability

Data for all experiments can be accessed here: https://osf.io/fj7de/.
