# Supplementary material for: Impaired grouping of ambient facial images in autism
Source: Sci Rep. 2022 Apr 23;12:6665. doi: 10.1038/s41598-022-10630-0 (PMC9035147; doi:10.1038/s41598-022-10630-0)
Supplement: Supplementary file 1 — Supplementary Information. [file 41598_2022_10630_MOESM1_ESM.docx]

|  | **Age** | **Sex** | **Gender identity** | **Ethnicity** | **AQ** | **PI20** | **TAS** | **IRI** | **ART** | **Exp 1** | **Exp 2** |
| --- | --- | --- | --- | --- | --- | --- | --- | --- | --- | --- | --- |
| 1 | 52 | Female | Female | White-British | 38 | 57 | 39 | 94 | 27 | 31 | 19 |
| 2 | 22 | Female | Female | Mixed ethnic group-White & Asian | 45 | 57 | 60 | 96 | 26 | 22 | 23 |
| 3 | 26 | Female | Non-binary | White-British | 33 | 85 | 64 | 85 | 29 | 25 | 29 |
| 4 | 50 | Female | Female | White-British | 47 | 78 | 86 | 84 | 24 | 25 | 24 |
| 5 | 44 | Female | Female | White-British | 44 | 92 | 71 | 94 | 29 | 35 | 24 |
| 6 | 23 | Female | Female | White-Irish | 39 | 92 | 57 | 104 | 25 | 26 | 25 |
| 7 | 20 | Male | Non-binary | White-British | 43 | 67 | 73 | 73 | 31 | 22 | 18 |
| 8 | 26 | Male | Male | White-British | 33 | 52 | 65 | 106 | 18 | 22 | 26 |
| 9 | 38 | Male | Male | White-British | 47 | 85 | 54 | 70 | 30 | 21 | 30 |
| 10 | 23 | Female | Male | White-British | 45 | 78 | 85 | 106 | 30 | 27 | 31 |
| 11 | 26 | Male | Male | White-British | 34 | 63 | 80 | 91 | 31 | 23 | 23 |
| 12 | 19 | Female | Female | White-British | 44 | 50 | 55 | 72 | 23 | 28 | 24 |
| 13 | 21 | Female | Female | White-British | 37 | 33 | 49 | 97 | 17 | 33 | 21 |
| 14 | 38 | Female | Female | White-British | 44 | 66 | 67 | 91 | 23 | 30 | 32 |
| 15 | 31 | Male | Male | White-British | 37 | 59 | 70 | 104 | 30 | 33 | 25 |
| 16 | 48 | Female | Female | White-British | 44 | 66 | 72 | 76 | 33 | 27 | 24 |
| 17 | 55 | Male | Male | White-British | 44 | 33 | 75 | 94 | 21 | 33 | 22 |
| 18 | 45 | Male | Male | White-British | 38 | 66 | 53 | 90 | 27 | 32 | 34 |
| 19 | 36 | Male | Male | White-British | 44 | 58 | 88 | 83 | 21 | 25 | 24 |
| 20 | 21 | Female | Non-binary | White-British | 41 | 63 | 84 | 92 | 22 | 30 | 26 |
| 21 | 35 | Male | Non-binary | White-British | 39 | 52 | 41 | 99 | 24 | 20 | 32 |
| 22 | 57 | Female | Non-binary | White-British | 47 | 69 | 79 | 68 | 20 | 25 | 33 |
| 23 | 45 | Male | Male | White-British | 44 | 81 | 79 | 93 | 28 | 17 | 35 |
| 24 | 28 | Male | Non-binary | White-British | 34 | 53 | 58 | 127 | 35 | 25 | 38 |
| 25 | 22 | Female | Non-binary | White-British | 37 | 55 | 68 | 105 | 35 | 29 | 27 |
| 26 | 34 | Male | Male | Other White | 42 | 55 | 48 | 117 | 30 | 28 | 28 |
| 27 | 43 | Female | Female | White-British | 38 | 83 | 66 | 102 | 18 | 26 | 26 |
| 28 | 34 | Male | Male | White-British | 41 | 73 | 60 | 90 | 16 | 27 | 23 |
| 29 | 21 | Female | Non-binary | White-British | 46 | 69 | 69 | 104 | 37 | 15 | 22 |
| 30 | 32 | Female | Female | White-British | 48 | 67 | 70 | 65 | 33 | 22 | 38 |
| 31 | 22 | Female | Female | White-British | 46 | 57 | 82 | 102 | 18 | 24 | 28 |
| 32 | 19 | Male | Male | Mixed ethnic group-Other Mixed | 46 | 59 | 74 | 54 | 18 | 18 | 25 |
| 33 | 21 | Female | Female | White-British | 41 | 41 | 69 | 82 | 15 | 29 | 30 |
| 34 | 33 | Female | Female | White-British | 41 | 67 | 82 | 71 | 16 | 22 | 32 |
| 35 | 54 | Female | Female | White-British | 42 | 74 | 70 | 74 | 28 | 25 | 30 |
| 36 | 21 | Male | Male | White-British | 33 | 81 | 70 | 95 | 25 | 24 | 27 |
| 37 | 23 | Female | Female | Other White | 46 | 58 | 36 | 85 | 22 | 36 | 24 |
| 38 | 25 | Female | Male | Mixed ethnic group-White & Black Caribbean | 39 | 40 | 57 | 94 | 31 | 34 | 25 |
| 39 | 21 | Female | Male | White-British | 47 | 69 | 70 | 107 | 22 | 25 | 17 |
| 40 | 43 | Male | Male | Other White | 43 | 66 | 56 | 107 | 33 | 20 | 28 |
| 41 | 19 | Female | Female | White-British | 35 | 57 | 36 | 98 | 26 | 23 | 33 |
| 42 | 24 | Female | Female | White-British | 35 | 57 | 57 | 106 | 26 | 23 | 31 |
| 43 | 33 | Female | Non-binary | White-British | 43 | 87 | 54 | 107 | 23 | 26 | 30 |
| 44 | 22 | Female | Female | White-British | 39 | 51 | 86 | 110 | 33 | 25 | 21 |
| 45 | 32 | Female | Non-binary | Mixed ethnic group-White & Black Caribbean | 43 | 67 | 64 | 99 | 30 | 25 | 31 |
| 46 | 43 | Female | Female | Other White | 38 | 60 | 68 | 99 | 23 | 29 | 37 |
| 47 | 54 | Female | Female | White-British | 41 | 79 | 65 | 87 | 29 | 24 | 16 |
| 48 | 20 | Female | Female | White-British | 41 | 63 | 71 | 108 | 33 | 29 | 25 |
| 49 | 26 | Male | Male | White-British | 41 | 63 | 62 | 99 | 26 | 30 | 24 |
| 50 | 48 | Male | Non-binary | Other White | 38 | 61 | 57 | 81 | 23 | 32 | 31 |
| 51 | 44 | Female | Female | White-British | 41 | 80 | 45 | 126 | 14 | 32 | 32 |
| 52 | 33 | Male | Female | White-British | 41 | 92 | 73 | 99 | 28 | 22 | 26 |
| 53 | 36 | Female | Female | White-British | 48 | 56 | 69 | 78 | 27 | 31 | 28 |
| 54 | 49 | Female | Female | White-British | 43 | 53 | 88 | 74 | 16 | 17 | 16 |
| 55 | 26 | Female | Female | White-British | 44 | 51 | 86 | 83 | 22 | 28 | 18 |
| 56 | 42 | Female | Female | Other White | 43 | 92 | 65 | 47 | 32 | 16 | 23 |
| 57 | 39 | Male | Prefer not to say | White-British | 45 | 65 | 71 | 80 | 29 | 32 | 23 |
| 58 | 25 | Male | Male | White-British | 38 | 93 | 91 | 76 | 20 | 30 | 21 |
| 59 | 25 | Male | Male | White-British | 32 | 30 | 62 | 115 | 25 | 37 | 32 |
| 60 | 28 | Female | Female | White-British | 46 | 86 | 83 | 82 | 31 | 31 | 36 |

| AQ = Autism Spectrum Quotient  PI20 = Twenty Item Prosopagnosia Index  TAS = Toronto Alexithymia Scale  IRI = Interpersonal Reactivity Index | ART = Abstract Reasoning Task  Exp 1 = Experiment 1  Exp 2 = Experiment 2 |
| --- | --- |
